# Aroma Rather than Taste Quality Exerted a More Pronounced Response to Organic Fertilizer Substitution in a Tea Garden: A Case Study on the Yellow Tea Quality

**DOI:** 10.3390/foods15101655

**Published:** 2026-05-09

**Authors:** Shenghong Zheng, Bo Zhu, Chunju Peng, Hongling Chai, Qi Huang, Zhengwen Niu, Ke Zhang, Guanghui Zeng, Xingjun Wen, Huajing Kang

**Affiliations:** 1 Wenzhou Key Laboratory of Early-Sprouting Tea Breeding, Wenzhou Academy of Agricultural Sciences, Wenzhou 325006, China; zsh1418@126.com (S.Z.); chunjupeng@163.com (C.P.); chaihl2021@163.com (H.C.); huangqi@wzvcst.edu.cn (Q.H.); niuzhengwen@wzvcst.edu.cn (Z.N.); zhangke@wzvcst.edu.cn (K.Z.); zengguanghui@wzvcst.edu.cn (G.Z.); 2 Pingyang County’s Science and Technology-Driven Agriculture Industrial Research Institute, Wenzhou 325006, China; 3 Uni-President China Holdings Ltd., Kunshan 215300, China; edwardzhu@pec.com.cn; 4 Lishui Wenji Tea Industry Co., Ltd., Lishui 323000, China

**Keywords:** organic fertilizer substitute, sensory quality, chemical components, aroma quality, Pingyang Huangtang tea

## Abstract

The warm and smooth taste, combined with the pleasant sweet aroma, are unique quality characteristics of Pingyang Huangtang (PYHT) tea. However, the potential to refine the quality through agricultural practices remains poorly explored. In this study, a one-year field experiment was conducted in the core production region of PYHT tea to assess the impacts of organic fertilizer substitution on tea quality. The experiment consisted of three fertilization regimes: T0 (pure chemical fertilizer), T1 (chemical fertilizer with rapeseed cake), and T2 (chemical fertilizer with newly organic fertilizer). Through sensory evaluation, determination of major biochemical components, and GC-MS analysis of aromatic compounds, combined with multivariate statistical methods, results showed that varied fertilization treatments significantly affected the aroma quality of PYHT tea, as indicated by T2 scoring the highest, surpassing the T0 and T1 scores, whereas no significant differences were observed in appearance, taste, liquor color, infused tea, or total score. Compared with chemical fertilization, organic fertilizer substitutes significantly reduced the total content of ammonia acids, a change primarily attributed to the decrease in the Thea, Arg, and Val levels, while the tea polyphenols and caffeine had no significant differences among treatments. GC-MS and multivariate analysis identified five key volatiles as octan-1-ol, linalool, geraniol, benzeneacetaldehyde, and δ-cadinene that differentiated the tea samples’ aroma profile. The distinct abundance patterns of these compounds are likely responsible for a fresher and more pronounced floral style of T2 tea, compared to the slightly less fresh floral notes of T0 tea and the stuffy floral expression of T1 tea. In summary, the research findings offer actionable guidelines for fertilizer application to PYHT tea production, improving aroma quality and increasing its overall market value.

## 1. Introduction

Tea is the most widely consumed non-alcoholic beverage globally [1]. According to processing methods, tea is commonly classified into six types: green tea, black tea, oolong tea, white tea, dark tea, and yellow tea (YT) [2]. Notably, YT is mainly produced in several Chinese provinces, including Hunan, Anhui, Zhejiang, Hubei, Sichuan, and Guangdong [3]. YT has garnered significant interest due to its pleasant fragrance and smooth flavor, along with its potential health advantages, which include antioxidant, anti-inflammatory, anti-obesity, and anti-cancer properties, regulation of metabolic syndrome, and modulation of gut microbiota [4]. Among YT varieties, Pingyang Huangtang (PYHT), originating from Pingyang County in Zhejiang Province, is recognized as a representative yellow tea in China. PYHT tea is widely renowned for its distinctive “three yellows and one fragrance” features (i.e., yellow dry tea, yellow tea infusion, yellow-infused leaves, and corn-like aroma), which contribute to its unique position in the tea industry [5]. The unique flavor profile of PYHT tea is intricately connected to the biochemical compounds of the tea leaves that contribute to its taste and aroma, which is largely determined by both the quality of the raw materials and the specific processing procedures. Although previous studies have focused on identifying quality-related compounds during production and in final tea products [6,7,8], the impact of fresh tea leaves on the quality characteristics of PYHT tea remains inadequately understood.

The quality attributes of fresh tea leaves are influenced by factors such as origin, cultivar, season, plucking standards, and agronomic practices [9,10]. Among these, fertilization serves as a key regulatory measure. Nutrient deficiency significantly reduces amino acid and aroma compound levels, with theanine content dropping to 11.4% of the control level [11], severely compromising quality. While excessive chemical nitrogen fertilizer can rapidly supplement nitrogen, long-term overuse inhibits the synthesis of polyphenols and flavonoids, leading to a bitter and astringent infusion [12]. Appropriate phosphorus application promotes root growth and energy metabolism, thereby facilitating the accumulation of sugars and aroma precursors and enhancing floral and fruity notes; however, high phosphorus levels suppress polyphenol accumulation [13,14]. Potassium, often termed the “quality element,” enhances metabolism and stress resistance, promotes amino acid conversion, contributes to a fresh, mellow, and harmonious taste, and improves the color of infused leaves [15]. Nevertheless, excessively high levels of phosphorus and potassium reduce the content of free amino acids such as theanine and glutamic acid while increasing flavonoid accumulation [16]. Therefore, a balanced application of nitrogen, phosphorus, and potassium is essential for comprehensively improving the color, aroma, and taste of tea.

Compared to the sole application of chemical fertilizers, organic fertilizers and their combined use with chemical fertilizers demonstrate significant advantages in enhancing tea quality and improving soil health [17]. Research indicates that organic fertilizers can effectively increase the levels of important biochemical components in tea, such as free amino acids and catechins, particularly when 30–50% of organic fertilizers replace chemical fertilizers, where the effects are especially pronounced [18]. Additionally, organic fertilizers can significantly lower the ratio of phenolic to amino acids and decrease the tea bitterness index, thereby improving flavor harmony [19]. In terms of sensory quality, organic fertilizers enhance the formation of aroma compounds, including linalool and geraniol, leading to a fresher and more vibrant tea aroma, as well as a more refreshing and mellow taste, while also enhancing the color and uniformity of the tea leaves [13,20]. Collectively, these effects indicate that organic fertilizers optimize the foundational composition of fresh tea leaves, thereby enhancing tea quality from chemical composition to sensory experience.

In tea plantations, rapeseed cake often serves as a high-quality organic fertilizer, with its core advantage being its high compatibility with the nutritional needs of tea trees [21,22]. It is rich in organic matter and contains significant amounts of nitrogen, phosphorus, and potassium, particularly high nitrogen content, which effectively promotes the growth of new shoots in tea trees [23]. In terms of quality enhancement, it leads to a significant increase in free amino acids and aromatic compounds in tea leaves, optimizing the phenolic to amino acid ratio, and is renowned as a “fragrance-enhancing fertilizer” [20,23,24]. Moreover, as a plant-based fertilizer, it allows for safe improvement of soil structure and creates a loose, acidic environment that aligns with the tea tree’s preference for acidic conditions, thereby achieving comprehensive benefits of increased yield, improved quality, and enhanced soil health [25]. However, existing studies have predominantly concentrated on the quality analysis of green tea, while research regarding the effects of various fertilization regimes on the YT quality is still inadequate. Thus, to investigate the influence of organic fertilizers on the quality of PYHT tea, we conducted a one-year field trial comparing organic substitutes with chemical fertilizers. We analyzed the effects of various fertilization treatments on both the sensory quality and key metabolites of PYHT tea. Notably, in addition to the rapeseed cake, we introduced a newly developed organic fertilizer specifically designed for tea plants, which adheres to the principle of high nitrogen, low phosphorus, and medium potassium ratios, featuring a total organic matter content exceeding 30% and an N:P_2_O_5_:K_2_O ratio of 20:3:10. The objective of this study is to elucidate the impact of organic fertilizers on the quality of PYHT tea, thereby providing a theoretical framework for optimal fertilization practices and enhancing tea quality in the Pingyang tea plantations. Furthermore, this study aims to offer practical guidelines for tea farmers to improve both the aromatic quality and overall market value of their tea.

## 2. Materials and Methods

### 2.1. Site Description and the Field Experiment

The field experiment was conducted in Pingyang County, Zhejiang Province, China (27°34′ N, 120°20′ E), with a mean annual temperature of 18.5 °C and annual precipitation of 1791 mm. This area is characterized by Ultisol soil with a loamy clay texture, which provides suitable edaphic conditions for tea cultivation. The tea cultivar was ‘Pingyang Tezao’ (*Camellia sinensis* L.), a clonal and early-maturing tea tree cultivar that serves as a high-quality raw material for the production of PYHT tea. Prior to the experiment, the properties of the surface soil (0–20 cm) were measured as follows: pH 4.11, soil organic carbon 5.67 g kg^−1^, total nitrogen 0.51 g kg^−1^, available phosphorus at 2.69 g kg^−1^, and available potassium at 37.60 g kg^−1^.

A randomized block design consisting of three treatments (i.e., T0, T1, and T2) with three replicates was established in 2024, resulting in a total of 9 plots. The experimental tea garden was arranged with an inter-row spacing of 1.3 m and a per-plot area of 15.6 m^2^, and a uniform nitrogen application rate of 300 kg N ha^−1^ was maintained across all treatments. T0 was set as the treatment with NPK pure chemical fertilizer (Sino-Arab Chemical Fertilizers Co., Ltd., Qinhuangdao, China). The chemical N fertilizers included urea and compound fertilizers (N:P:K = 17:17:17), with the latter also providing phosphorus (P) and potassium (K) nutrients. The annual application rates of phosphorus and potassium were maintained at 150 kg P_2_O_5_ ha^−1^ and 150 kg K_2_O ha^−1^, respectively. T1 treatment derived 50% of its nitrogen from chemical fertilizers and the remaining 50% from rapeseed cake (Pingyang County Oasis Ecological Bioengineering Co., Ltd., Wenzhou, China). Equal amounts of nitrogen (150 kg N ha^−1^) were provided by each source. The application rate of rapeseed cake was determined based on its N content of 5%. For the chemical fertilizers, both urea (Huiduoli Agricultural Materials Co., Ltd., Hangzhou, China) and compound fertilizers were applied, each contributing 50% of the N from chemical sources. The application rate for urea was calculated according to its nitrogen content of 46.67%, while the application rate for the compound fertilizer was based on a N content of 17%. Similarly, T2 treatment was designed to consist of 50% N sourced from chemical fertilizers and 50% from newly organic fertilizers manufactured by Inner Mongolia Ruijiatian Agricultural Technology Development Co., Ltd (Wulanchabu, China). Specifically, chemical fertilizers contributed 150 kg N ha^−1^, while an additional 150 kg N ha^−1^ was provided by newly organic fertilizers. The application rate for newly organic fertilizers was determined based on an N content of 1.23%. During the experiment, compound fertilizer, rapeseed cake, and newly organic fertilizer were applied as base fertilizers, while urea was administered in three stages: summer topdressing (accounting for 20% of the total application, applied in early May 2024), base fertilizer (50%, addressed in early November 2024), and spring topdressing (30%, conducted in early February 2025). All fertilizers were applied in 20 cm-deep furrows and subsequently covered with soil.

### 2.2. Sample Preparation

**Figure 1:**
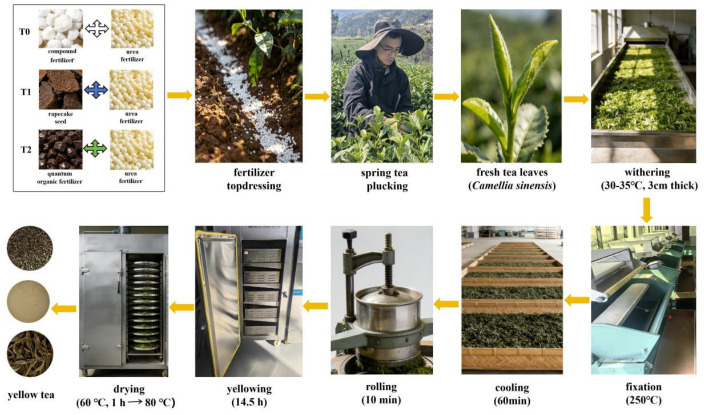
The manufacturing flowchart of PYHT tea with fresh tea leaves from varied fertilization regimes.

In March 2025, newly sprouting shoots with one bud and one leaf were plucked. PYHT tea was then processed using a standard manufacturing procedure, including withering, fixation, cooling, rolling, sealing, yellowing, and drying (Figure 1). The shoots were withered for 14 h, then fixed in a flat tea-frying machine at 250 °C until the moisture content of the fixed shoots reached approximately 65%, consistent with the local production practice of PYHT tea. After cooling at room temperature for 1 h, the leaves were gently rolled for about 10 min until slight curling occurred. The rolled leaves were then subjected to sealed yellowing in a wrapped bag at 40 °C for 14.5 h. Finally, the yellowed leaves were dried at 60 °C for 1 h, followed by 80 °C for about 2.5 h until the moisture content dropped below 6%. All tea samples were used for sensory evaluation, while a portion was ground into a powder in a grinding mill and used for subsequent physicochemical analysis.

### 2.3. Sensory Evaluation

A sensory panel consisting of five trained experts (graded II or above) qualified in tea sensory evaluation was assembled in accordance with the Tea Sensory Evaluator (GZB 6-02-06-11, [26]) standards. Sensory assessment was conducted following the Methodology for Sensory Evaluation of Tea (GB/T 23776-2018, [27]) and the Tea Vocabulary for Sensory Evaluation (GB/T 14487-2017, [28]). In summary, dry tea appearance was first evaluated and scored based on its color and shape in the sample tray. Subsequently, 3 g of the yellow tea samples were infused with 150 mL of boiling water in a covered teacup for 5 min, after which the infusion was filtered into a corresponding tea bowl. The color, aroma, taste of infusion, and the characteristics of the infused leaves were evaluated, described, and scored sequentially. A 100-point scoring system was employed for the evaluation, with weighting assigned as follows: appearance (25%), infusion color (10%), aroma (25%), taste (30%), and infused leaves (10%). Ethical approval was not required, and all panelists provided informed consent to participate and to have their data used. The purified water used for sensory evaluation was purchased from Wahaha Group Corporation (Hangzhou, China).

### 2.4. Determination of Non-Volatile Compounds

#### 2.4.1. Chemicals and Standards

Epicatechin (EC), (+) catechin (C), epigallocatechin (EGC), epigallocatechin gallate (EGCG), gallocatechin gallate (GCG), epicatechin gallate (ECG), gallocatechin (GC), caffeine, were obtained from Sigma-Aldrich Corporation (St. Louis, MO, USA). High-performance Liquid Chromatographic (HPLC) grade acetonitrile and methanol were supplied by Merck Inc. (Shanghai, China). Formic acid, acetic acid (HPLC grade), and deuterated guaiacol (≥99% purity) were provided by Aladdin Inc. (Shanghai, China). Ethanol, disodium hydrogen phosphate, potassium dihydrogen phosphate, folin-ciocalteureagent, ninhydrin hydrate, stannous chloride, sodium carbonate, and glutamic acid were analytical-grade reagents purchased from Sinopharm Chemical Reagent Co., Ltd. (Shanghai, China). The ultrapure water generated using a Barnstead™ GenPure™ water system (Thermo Fisher Scientific, Waltham, MA, USA) was used for the tea component.

#### 2.4.2. Determination of Main Taste Quality Components

The tea infusion samples were prepared by brewing tea samples with boiling water at a tea-to-water ratio of 1:50 (*w*/*w*) for 4 min.

The total polyphenols (TPs) of the tea samples were quantified using the Folin–Ciocalteu colourimetric method, as outlined in GB/T 8313-2018, [29]. Briefly, 1 mL of the sample or gallic acid (GA) standard (0–100 mg/L) was transferred into a 10 mL volumetric flask, followed by the addition of 5 mL of Folin–Ciocalteu reagent (10%, *v*/*v*) and 4 mL of sodium carbonate (Na_2_CO_3_) solution (7.5% *v*/*v*). The resulting mixture was incubated at room temperature for 60 min, after which the absorbance was subsequently recorded at 765 nm using a UV-Vis spectrophotometer (UV3600, Shimadzu, Tokyo, Japan).

Free amino acids (FAAs) in the tea samples were quantified using the ninhydrin colorimetric method according to GB/T 8314-2013, [30]. 1 mL of tea sample or glutamic acid standard (0–0.9 mg/mL) was mixed with 0.5 mL of phosphate buffer (pH 8.0) and 0.5 mL of a 2% ninhydrin solution in a 25 mL volumetric flask. The mixture was then heated in a boiling water bath at 100 °C for 15 min and subsequently cooled to a constant volume. Absorbance was recorded at 570 nm after standing for 10 min.

To determine the FAA component content, 0.2 g of dried tea powder was extracted with 10 mL of ultrapure water at a constant temperature of 100 °C for 30 min. The extracts were shaken for 10 min and centrifuged at 3500 rpm for 10 min, and the supernatant was collected and filtered through a 0.22 μm membrane filter (Membrane Solutions, Kent, WA, USA) for further analysis. Free amino acids were derivatised using an AccQ-Fluor Reagent Kit (Waters, Milford, MA, USA) according to the manufacturer’s specifications. 10 μL of standard amino acid mixture or tea extract was mixed with 70 μL of AccQ Tag borate buffer and 20 μL of AccQ Tag reagent pre-dissolved in 1.0 mL of AccQ Tag reagent diluent, followed by incubation at 55 °C for 10 min. Separation was carried out on an HPLC system (Waters, Milford, MA, USA) equipped with a Waters AccQ Tag reversed-phase HPLC column (150 mm × 3.9 mm, 4 μm). Mobile phase A was composed of AccQ Tag Eluent A Concentrate diluted in deionized water (1:10 *v*/*v*), while mobile phase B consisted of acetonitrile, and mobile phase C was ultrapure water. The total running time was 45 min, with a sample injection volume of 5 μL and a flow rate of 1.0 mL/min. The column temperature was maintained at 37 °C, and amino acids were detected at 248 nm, identified by comparing retention times and spectra with standard solutions of the amino acid kit and L-theanine.

Catechins and caffeine were extracted from the tea sample using 50% aqueous ethanol and quantified by an HPLC method previously reported [31]. Specifically, 0.2 g of dried tea sample was extracted with 5 mL of 70% methanol in a 70 °C water bath for 10 min with intermittent shaking. The supernatant was transferred to a 10 mL volumetric flask, and the extraction was repeated to a final volume of 10 mL. The extracts were filtered through a 0.45-μm Millipore filter prior to injection. All experiments were performed in triplicate. The extracts were analyzed using an Agilent 1100 HPLC system (Agilent Technologies Inc., Santa Clara, CA, USA). Separations were achieved on a Phenomenex RP-MAX 4 μm 250 mm × 4.6 mm i.d. C12 reverse-phase column (Phenomenex, Inc., Torrance, CA, USA) maintained at 40 °C, with a flow rate of 1 mL min^−1^ and a 60 min gradient of 4–25% acetonitrile in water containing 1% formic acid, monitored at 280 nm [32]. Caffeine, catechin (C), epicatechin gallate (ECG), epigallocatechin (EGC), epigallocatechin gallate (EGCG), gallocatechin gallate (GCG), and epicatechin (EC) were identified by comparing their retention times with those of standard solutions. The relative retention time of each HPLC peak was calculated using the largest peak (EGCG) as the reference. For enhanced accuracy, unidentified peaks were carefully verified by matching both the absolute retention time and the relative retention time of each peak in the samples. Matched peaks that consistently appeared in the samples were utilized as fingerprints to distinguish geographic origins.

### 2.5. Detection of Volatiles

The tea samples were ground to a powder, and 1.5 g of each sample was placed in a 20 mL headspace vial. Subsequently, 5 mL of a 25% sodium chloride solution containing 1/60,000 n-octanol (*v*/*v*) as an internal standard was added to the sample. The vial was immediately sealed with a cap, and the protective cap was pierced with a DVB/CAR/PDMS fiber tip attached to a manual handle (SPME, Supelco, PA, USA). Finally, the vial was incubated on a constant-temperature heating plate at 65 °C for 1 h to allow adsorption of aroma compounds.

The volatile aroma compounds were analyzed using a GC-MS/MS system (Agilent Technologies 7890B GC-MS). The chromatographic column used was an HP-5MS (30 m × 0.25 mm, 1909IS-433UI, Agilent Technologies Inc., CA, US). For the GC-MS analysis, the injection temperature was maintained at 250 °C, and a temperature gradient of 5 °C/min was applied, starting at 40 °C (held for 3 min) and reaching 250 °C. The electron impact (EI) Ionization mode was operated at 70 eV. Mass spectrometry data were collected over a range of 40 to 400 *m*/*z* [33,34]. Qualitative analysis was performed using the Agilent MassHunter Unknowns analysis program to identify the compounds. The aroma compounds were characterized by matching their spectra with the NIST 17.0 library and confirming them by comparing their retention indices (RI). Quantification was achieved by calculating the ratio of the peak area of each detected volatile compound to that of the internal standard (n-octanol) for relative quantification. The estimation formula, as reported in our previous study [35], is as follows:

$$X_{i}=\frac{V_{s}\;\times \;C_{s}}{M}\;\times \;\frac{I_{i}}{I_{s}}\;\times {\;10}^{-3}$$

where X_i_ represents the concentration of compound i in the sample (μg/g); V_s_ is the volume of the internal standard added (μL); C_s_ denotes the concentration of the internal standard (μg/mL); M is the sample mass (1500 mg); I_s_ is the peak area of the internal standard; I_i_ is the peak area of compound i in the sample.

The ratio of the target volatile’s relative content (Ci) to its odor threshold (OT) was calculated to derive the relative odor activity values (rOAVs) for the target. This approach was used to evaluate the contribution of each volatile to the overall aroma profile of the samples. Generally, a volatile is considered a potential contributor to aroma when its rOAV exceeds 1.

### 2.6. Data Analysis and Figure Preparation

All data were presented as mean ± standard deviation (SD) based on three replicates. Statistical analysis was performed using one-way analysis of variance (ANOVA) in SPSS 25 (IBM, Armonk, NY, USA), and differences were considered statistically significant at *p* < 0.05. Principal component analysis (PCA) and orthogonal partial least squares discriminant analysis (OPLS-DA) were conducted in SIMCA 14.1 (Umetrics, Umea, Sweden) to assess the overall distribution among the tea samples and the stability of the entire process. Variable Importance in Projection (VIP) scores were used to rank the contribution of each variable to group discrimination, as derived from the OPLS-DA. Differential metabolites were identified with VIP scores greater than 1.0 and *p*-values less than 0.05. Heatmaps and bar graphs were generated in Origin2025b (OriginLab, Northampton, MA, USA).

## 3. Results

### 3.1. Sensory Evaluation of PYHT Tea

**Figure 2:**
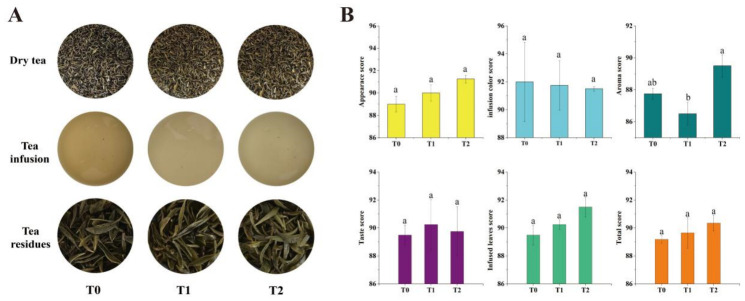
Results of sensory evaluation of PYHT teas. (**A**) Appearance of dry tea, tea liquor, and infused leaves. (**B**) Sensory scores. All values are presented as mean ± SD, and different lowercase letters (a and b) indicate significant differences at *p* < 0.05.

The results of sensory evaluation indicate that PYHT tea produced from fresh leaves treated with organic fertilizer substitutes exhibited higher overall quality than that of leaves treated solely with chemical fertilizers. The PYHT tea treated with pure chemical fertilizers exhibited tender yellow leaves tinged with brown, appearing slightly dull, accompanied by a relatively bright yellow liquor and yellowish, somewhat bright brewed leaves. In comparison, the PYHT tea treated with rapeseed cake substitute (T1) displayed tender, yellow, and bright leaves with good moisture, light-yellow and bright liquor, and yellowish, bright brewed leaves. Furthermore, the newly organic fertilizer-treated yellow tea (T2) presented tender yellow and bright leaves with a moist appearance, light yellow and luminous liquor, and yellowish, fresh-looking brewed leaves (Figure 2A). The sensory scoring results showed that the mean appearance scores of T0, T1, and T2 were 89 ± 0.71, 90 ± 0.71, and 91.25 ± 0.35, respectively. For liquor color, T0 exhibited the highest score of 92 ± 2.83, slightly higher than T1 tea’s 91.75 ± 1.77 and T2 tea’s 91.50 ± 0.71. T2 showed the highest aroma score of 89.5 ± 0.71, exceeding T0 tea’s 87.75 ± 0.35 and being significantly (*p* < 0.05) higher than T1 tea’s 86.5 ± 0.71. Regarding taste quality, T1 scored the highest in flavor at 90.25 ± 1.77, insignificantly higher than T2 tea’s 89.75 ± 1.77 and T0 tea’s 89.50 ± 0.71. In terms of infused leaves, T2 recorded the highest score of 91.50 ± 0.71, exceeding T1 tea’s 90.25 ± 0.35 and T0 tea’s 89.50 ± 0.71. Similarly, T2 also scored the highest overall at 90.35 ± 0.57, which was non-significantly higher than T1 and T0 teas at 89.65 ± 1.10 and 89.19 ± 0.27, respectively (Figure 2B).

### 3.2. Effect of Different Fertilization Treatments on Taste-Related Components of PYHT Tea

**Figure 3:**
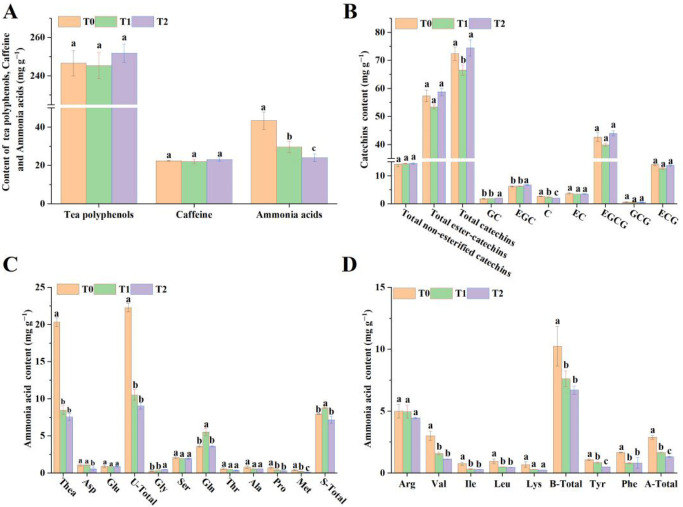
Taste-related components in PYHT teas produced from fresh leaves under different fertilization regimes. (**A**) Content of tea polyphenols, caffeine and ammonia acids; (**B**) content of tea catechins; (**C**) content of umami and sweet ammonia acids, U-total denotes total content of umami ammonia acids and S-total indicates total content of sweet ammonia acids; (**D**) content of bitterness and aromatic ammonia acids, B-total means total content of bitter ammonia acids and A-total represents the total content of aromatic ammonia acids. All values are presented as means ± SD, and different lowercase letters (a, b, and c) indicate significant differences at *p* < 0.05.

Overall, the application of organic substitution in tea gardens led to a significant reduction in total free amino acid content in yellow tea. The levels observed under rapeseed cake and newly organic fertilizer treatments were significantly lower than those in yellow tea treated solely with chemical fertilizers. Furthermore, in comparison to treatments using pure chemical fertilizers, organic substitution treatments exhibited minimal influence on the total contents of tea polyphenols and caffeine in yellow tea. The contents of tea polyphenols in T0, T1, and T2 yellow teas were consistent, and caffeine levels remained stable, with no significant differences observed (Figure 3A).

Further analysis of catechins showed that, compared with the pure chemical fertilizer treatment (T0), organic substitution treatments increased the levels of two non-esterified catechins (GC and EGC) in yellow tea. Among them, yellow tea treated with quantum organic fertilizer (T2) exhibited significantly higher GC and EGC levels than those treated with rapeseed cake fertilizer (T1) and pure chemical fertilizer (T0) (Figure 3B). Meanwhile, organic substitution treatments significantly reduced the catechin (C) content in yellow tea. The C content in T0 was 2.68 ± 0.10 mg g^−1^, significantly higher than that in T1 (2.42 ± 0.05 mg g^−1^) and T2 (2.07 ± 0.03 mg g^−1^). Under organic substitution treatments, the EC content in yellow tea was slightly lower than in T0, while the total content of non-esterified catechins was slightly higher than in T0, but neither difference reached statistical significance. Regarding ester-type catechins, the contents of EGCG, GCG, ECG, and total esterified catechins were all lowest in yellow tea treated with rapeseed cake (T1), while the contents in T0 and T2 were similar, with no significant differences. Statistical analysis also revealed that the total catechin content was highest in T2 yellow tea, followed by T0, both of which were significantly higher than in T1 yellow tea (Figure 3B).

Analysis of amino acid components showed that organic substitution treatments significantly reduced the content of theanine (Thea) and total umami amino acids in yellow tea, while moderately decreasing the levels of two umami amino acids, aspartic acid (Asp) and glutamic acid (Glu) (Figure 3C). Regarding sweet-tasting amino acids, compared with T0, organic substitution treatments increased the contents of glycine (Gly) and glutamine (Gln) in yellow tea. Specifically, the T1 treatment significantly enhanced Gln content, while the T2 treatment notably increased Gly content. Additionally, organic substitution treatments reduced the contents of serine (Ser), threonine (Thr), alanine (Ala), proline (Pro), and methionine (Met) to varying degrees. The T1 treatment showed the highest total content of sweet-tasting amino acids (8.78 ± 0.04 mg g^−1^), significantly surpassing both T0 (7.94 ± 0.05 mg g^−1^) and T2 (7.17 ± 0.45 mg g^−1^) treatments (Figure 3C). Concerning bitter and aromatic amino acids, compared with T0, organic substitution treatments showed a decreasing trend in the contents of all relevant amino acid monomers in yellow tea. The contents of three bitter amino acids—valine (Val), isoleucine (Ile), and leucine (Leu)-as well as two aromatic amino acids—tyrosine (Tyr) and phenylalanine (Phe), along with the total amounts of bitter amino acids and aromatic amino acids, were all significantly lower in the organic substitution treatment compared to the T0 treatment (Figure 3D).

### 3.3. Effect of Different Fertilization Treatments on the Volatiles of PYHT Teas

#### 3.3.1. Identification of Volatile Compounds in PYHT Tea

**Figure 4:**
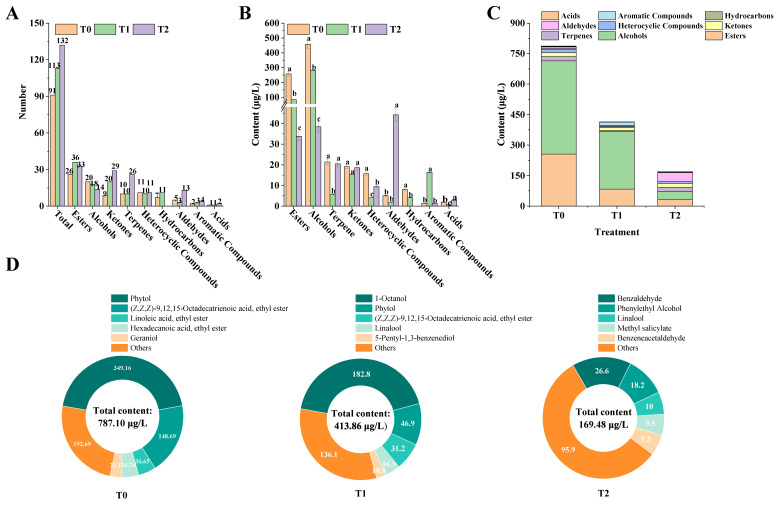
Analysis of aroma components in PYHT teas manufactured from fresh leaves under different fertilization regimes. (**A**) Number of volatile categories in yellow teas from T0, T1, and T2. (**B**) Comparison of volatile category contents in yellow teas from T0, T1, and T2. (**C**) Total content of aroma categories in yellow teas from T0, T1, and T2. (**D**) The top five most abundant aroma compounds in yellow teas from T0, T1, and T2. All values are presented as mean ± SD, and different lowercase letters (a, b, and c) indicate significant differences at *p* < 0.05.

A total of 91 volatile compounds (including 26 esters, nine ketones, 20 alcohols, 10 terpenes, 11 heterocyclic compounds, seven hydrocarbons, five aldehydes, two aromatic compounds and one acid) were detected in T0 tea, while 112 volatile compounds (including 36 esters, 20 ketones, 18 alcohols, 10 terpenes, 10 heterocyclic compounds, 11 hydrocarbons, three aldehydes, three aromatic compounds and one acid) were detected in T1 tea, and 132 volatile compounds (including 33 esters, 29 ketones, 14 alcohols, 26 terpenes, 11 heterocyclic compounds, 13 aldehydes, four aromatic compounds and two acids) were detected in T2 by HS-SPME-GC-MS (Figure 4A).

Alcohols were the predominant volatile compounds, with the highest concentrations in T0 tea (457.63 ± 26.22 µg/L) and T1 tea (282.01 ± 1.92 µg/L), respectively. By contrast, aldehydes were the primary volatile compounds in T2 tea, reaching 44.22 ± 2.99 µg/L (Figure 4B,C). Furthermore, T1 tea exhibited the lowest concentrations of terpenes, ketones, heterocyclic compounds, aldehydes, and acids, all of which were significantly lower than those in T0 and T2 teas. Conversely, T1 tea displayed the highest concentration of aromatic compounds, which was significantly greater than in T0 and T2 (Figure 4B). In terms of total volatile content, T0 tea had the highest concentration at 787.11 ± 45.10 µg/L, far exceeding the levels in T1 and T2 teas, which were measured at 426.74 ± 2.90 µg/L and 169.48 ± 11.46 µg/L, respectively (Figure 4C).

Further analysis of the aroma components in the three PYHT teas revealed that the five predominant aroma compounds in T0 tea were phytol, (Z,Z,Z)-9,12,15-octadecatrienoic acid ethyl ester, linoleic acid ethyl ester, hexadecanoic acid ethyl ester, and geraniol, with concentrations of 349.16 ± 20.01 µg/L, 148.69 ± 8.52 µg/L, 36.65 ± 2.10 µg/L, 36.74 ± 2.11 µg/L, and 23.17 ± 1.33 µg/L, respectively. In T1 tea, the top five aroma compounds were 1-octanol, phytol, (Z,Z,Z)-9,12,15-octadecatrienoic acid ethyl ester, linalool, and 5-pentyl-1,3-benzenediol, with concentrations of 182.79 ± 1.24 µg/L, 46.87 ± 0.32 µg/L, 31.22 ± 0.21 µg/L, 16.92 ± 0.11 µg/L, and 12.88 ± 0.99 µg/L, respectively. For T2 tea, the top five aroma compounds were benzaldehyde, phenylethyl alcohol, linalool, methyl salicylate, and benzeneacetaldehyde, with concentrations of 26.61 ± 1.80 µg/L, 18.20 ± 1.23 µg/L, 10.02 ± 0.68 µg/L, 9.50 ± 0.64 µg/L, and 9.27 ± 0.63 µg/L, respectively (Figure 4D).

#### 3.3.2. PCA and OPLS-DA Analysis in the Volatile Compounds

**Figure 5:**
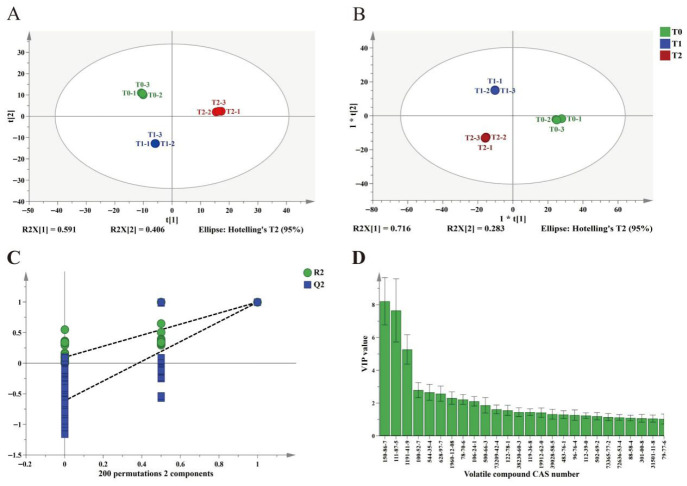
PCA and OPLS-DA analysis of PYHT in varied fertilization regimes. (**A**) PCA score plot. (**B**) OPLS-DA score plot. (**C**) Variable importance values above one in OPLS-DA modeling. (**D**) 200 permutation tests. “*” in (**B**) indicates that the axis was automatically scaled by the software for better visualization, with no other special statistical meaning (not *p*-value, not significance). CAS number corresponds with the volatile compound as follows: 150-86-7 = Phytol, 111-87-5 = octan-1-ol, 1191-41-9 = ethyl (9Z,12Z,15Z)-octadeca-9,12,15-trienoate, 100-52-7 = Benzaldehyde, 544-35-4 = ethyl (9Z,12Z)-octadeca-9,12-dienoate, 628-97-7 = Ethyl hexadecanoate, 1960-12-08= 2-Phenylethanol, 78-70-6 = Linalool, 106-24-1 = Geraniol, 500-66-3 = 5-pentylbenzene-1,3-diol,73209-42-4 = (1R,4S)-1,6-dimethyl-4-propan-2-yl-1,2,3,4-tetrahydronaphthalene,122-78-1 = Benzeneacetaldehyde,38230-60-3 = (1S,4S,6S,7S,10S)-4,10-dimethyl-7-propan-2-yltricyclo [4.4.0.01,5]dec-8-en-4-ol, 119-36-8 = Methyl salicylate, 19912-62-0 = (1S,4S,4aR,8aS)-1,6-dimethyl-4-propan-2-yl-3,4,4a,7,8,8a-hexahydro-2H-naphthalen-1-ol,39028-58-5 = (3R,6S)-6-ethenyl-2,2,6-trimethyloxan-3-ol,483-76-1 = (1S,8aR)-4,7-dimethyl-1-propan-2-yl-1,2,3,5,6,8a-hexahydronaphthalene,96-76-4 = 2,4-ditert-butylphenol,112-39-0 = methyl hexadecanoate,502-69-2 = 6,10,14-trimethylpentadecan-2-one,73365-77-2 = (1S,4S,4aS,8aR)-4,7-dimethyl-1-propan-2-yl-2,3,4,5,6,8a-hexahydro-1H-naphthalen-4a-ol,72636-53-4 = 2,5-dibutylfuran,88-58-4 = 2,5-ditert-butylbenzene-1,4-diol,301-00-8 = methyl (9Z,12Z,15Z)-octadeca-9,12,15-trienoate, 31501-11-8 = [(Z)-hex-3-enyl]hexanoate,79-77-6 = (E)-4-(2,6,6-trimethylcyclohexen-1-yl)but-3-en-2-one.

Unsupervised principal component analysis (PCA) was conducted on the volatile compounds identified by GC-MS. As shown in Figure 5A, the three PYHT teas under different fertilization regimes were clearly distinguished, with model parameters of R^2^X = 0.997 and Q^2^ = 0.995. Subsequently, supervised orthogonal partial least squares discriminant analysis (OPLS-DA) was performed using the PCA results, yielding model parameters of R^2^Y = 0.999 and Q^2^ = 0.998. Figure 5B shows that the three PYHT teas were clearly separated, indicating significant differences in the volatile compounds across the various organic fertilization substitute models. To assess model robustness, 200 permutation tests were conducted. The results showed that R^2^ and Q^2^ ranged from (0, 0.097) and (0, −0.615), respectively, indicating that the model was robust and free from overfitting (Figure 5C).

The Variable Importance in Projection (VIP) scores in the OPLS-DA model quantify the differential contributions of compounds present in the samples. A VIP value greater than 1 indicates that a compound substantially contributes to the observed differences between the samples. Through OPLS-DA analysis, we identified 26 volatile compounds with VIP values greater than 1 (Supplementary Materials, Figure 5D). These compounds included phytol, octan-1-ol, benzaldehyde, ethyl (9Z,12Z)-octadeca-9,12-dienoate, ethyl hexadecanoate, 2-phenylethanol, linalool, geraniol, methyl salicylate, and ethyl (9Z,12Z,15Z)-octadeca-9,12,15-trienoate, among others (Figure 5D).

#### 3.3.3. Analysis of Differential Volatile Compounds in PYHT Teas

**Figure 6:**
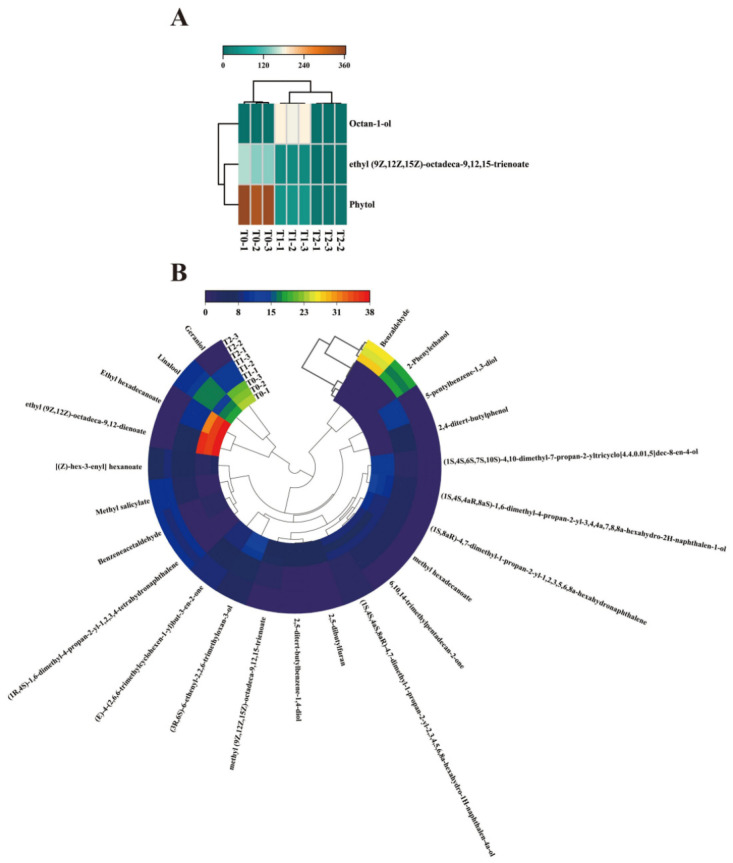
Heatmap constructed with 26 key differential volatiles with VIP > 1.0 in PYHT teas. (**A**) Three aroma compounds with maximum concentration exceeding 100 mg/g. (**B**) Twenty-three aroma compounds with concentrations below 40 mg/g.

To visualize the differences among the PYHT teas, a heatmap based on 26 key differential volatiles (VIP > 1.0) in PYHT teas was constructed, and the results are illustrated in Figure 6. Clearly, the brown and red color in the squares indicates higher content, while the green and blue indicate lower content. Three components exhibited maximum contents exceeding 100 mg/g, with phytol showing the highest concentration in T0 at 349.16 ± 14.15 µg/L, significantly surpassing T1 tea at 46.87 ± 0.23 µg/L and T2 tea at 10.08 ± 0.49 µg/L. Octan-1-ol was exclusively detected in T1 yellow tea, with a content of 182.79 ± 0.88 µg/L. Additionally, ethyl (9Z,12Z,15Z)-octadeca-9,12,15-trienoate was found at a higher level in T0 tea compared to T1 tea, while it was absent in T2 tea (Figure 6A). Moreover, ethyl (9Z,12Z)-octadeca-9,12-dienoate, geraniol, ethyl hexadecanoate,(1S,4S,6S,7S,10S)-4,10-dimethyl-7-propan-2-yltricyclo [4.4.0.01,5]dec-8-en-4-ol,(1S,4S,4aR,8aS)-1,6-dimethyl-4-propan-2-yl-3,4,4a,7,8,8a-hexahydro-2H-naphthalen-1-ol,methylhexadecanoate,δ-cadinene,(3R,6S)-6-ethenyl-2,2,6-trimethyloxan-3-ol,6,10,14-trimethylpentadecan-2-one,(1S,4S,4aS,8aR)-4,7-dimethyl-1-propan-2-yl-2,3,4,5,6,8a-hexahydro-1H-naphthalen-4a-ol,2,5-dibutylfuran, 2,5-ditert-butylbenzene-1,4-diol, methyl (9Z,12Z,15Z)-octadeca-9,12,15-trienoate, demonstrated higher levels in T0 compared to T1 and T2 teas. In contrast, three volatile compounds, including linalool, 5-pentylbenzene-1,3-diol, and 2,4-ditert-butylphenol, exhibited greater concentrations in T1 tea than in T0 and T2 teas. Compared to T0 and T1 teas, T2 showed elevated levels of seven compounds, including 2-phenylethanol, (1R,4S)-1,6-dimethyl-4-propan-2-yl-1,2,3,4-tetrahydronaphthalene, benzaldehyde, benzeneacetaldehyde, methyl salicylate, [(Z)-hex-3-enyl] hexanoate and (E)-4-(2,6,6-trimethylcyclohexen-1-yl)but-3-en-2-one (Figure 6B).

#### 3.3.4. rOAV Analysis of Volatile Compounds in PYHT Teas

rOAV is an important parameter for evaluating the contribution of volatile compounds to overall aroma. Generally, volatile compounds with an rOAV ≥ 1 are considered key contributors to the entire aroma profile. In this study, our analysis was performed based on previously reported aroma component thresholds and attribute descriptions in the literature [36,37,38]. The rOAV values of 26 differential aroma components in PYHT teas were calculated, and 5 differential aroma compounds with rOAV ≥ 1 were retained for further analysis (Table 1). Among the analyzed compounds, octan-1-ol was exclusively detected in the T1 tea with a rOAV of 8.31 ± 0.04, in contrast to the T0 and T2 teas. Linalool exhibited comparable rOAV values in T0 and T1 teas, recorded at 2.80 ± 0.38 and 2.82 ± 0.01, respectively, both significantly higher than the rOAV in T2 tea (1.68 ± 0.08). Geraniol demonstrated the highest rOAV in T0 tea (3.41 ± 0.22), which was significantly greater than that in T1 tea (1.66 ± 0.03), while it was not detected in T2 tea. Similarly, (1S,8aR)-4,7-dimethyl-1-propan-2-yl-1,2,3,5,6,8a-hexahydronaphthalene exhibited an elevated rOAV in T0, significantly surpassing that in T1 tea, with no detection in T2 tea. Furthermore, Benzeneacetaldehyde presented a rOAV exceeding one exclusively in T2 tea compared to T0 and T1 teas (Table 1). Subsequent analysis indicated that δ-cadinene had the highest aroma contribution index (ACI) in T0 tea (47.90%), followed by geraniol (27.54%), linalool (22.62%), and benzeneacetaldehyde (1.94%). In T1 tea, octan-1-ol ranked first in ACI (56.15%), followed by linalool (19.05%), δ-cadinene (12.77%), geraniol (11.22%), and benzeneacetaldehyde (0.81%). Additionally, linalool and geraniol were the only two key differential volatile compounds identified in T2 tea, with ACI values of 53.50% and 46.50%, respectively (Table 1).

## 4. Discussion

### 4.1. Impact of Organic Substitutes on the Sensory Quality of PYHT Teas

Sensory evaluation demonstrated that compared to the pure chemical fertilizer treatment (T0), both organic substitution treatments (T1 and T2) enhanced the color brightness of yellow tea’s dry leaves, liquor, and infused leaves. However, the yellowness of their liquor was inferior to that of T0 (Figure 2A). Studies indicate that extensive chlorophyll degradation coupled with carotenoid retention during the yellowing process is pivotal in forming the characteristic “yellow” tone of yellow tea’s dry leaves and infused leaves [3]. It can be inferred that carotenoid content governs the intensity of yellow coloration—higher content yields more pronounced yellowness, while chlorophyll content influences color brightness. Greater chlorophyll levels may produce more dark-colored degradation byproducts, potentially leading to duller hues in dry leaves and infused leaves. Further speculation suggests that in this study, the chlorophyll and carotenoid contents in fresh leaves treated with organic fertilizer were lower than those in chemical fertilizer treatments. After undergoing the same yellowing process, the chlorophyll degradation products in T1 and T2 were lower than those in T0, resulting in their dried tea and infused leaves exhibiting similar yellowing degrees but higher brightness than T0. Consequently, their corresponding scores were also higher than T0, aligning with the sensory evaluation requirements and results for yellow tea (Figure 2A). This conclusion is consistent with Hua’s findings, which revealed that tea fresh leaves treated with chemical fertilizer had higher chlorophyll and carotenoid contents than organic fertilizer treatments, along with a lower chlorophyll/carotenoid ratio, leading to significantly yellower dried tea and infused leaves in chemical fertilizer-produced green tea [39]. Additionally, the liquor color of T0 yellow tea was notably yellower than that of T1 and T2, indicating that T0 liquor contained more abundant water-soluble yellow pigments. Previous studies have indicated that the color substances in Pingyang Huangtang’s liquor primarily originate from water-soluble pigments formed through the transformation of catechins, such as theaflavins and catechin dimers [40]. It is thus inferred that pure chemical fertilizer treatment may promote the formation of catechin polymers like theaflavins, resulting in a more prominent yellow hue in the liquor. In terms of flavor, organic fertilizer treatments (T1, T2) generally improved the taste of yellow tea, with higher flavor scores than T0, although the differences were not statistically significant (Figure 2B). This trend aligns with findings from related studies on green tea [39]. Notably, significant differences in aroma quality of yellow tea were observed among different fertilization treatments, and aroma was the only sensory attribute that reached a significant difference (Figure 2B). The scores showed T2 > T0 > T1. This indicates that the aroma of yellow tea is particularly sensitive to fertilization methods, and different organic fertilizer treatments have distinct effects on the aroma, which is well supported by the previous studies [13,20,41]. This is also the main reason for subsequently focusing on the analysis of aroma components.

### 4.2. Impact of Organic Substitutes on the Taste-Related Components in PYHT Teas

This study analyzed the effects of three fertilization regimes on key quality components of yellow tea. The results indicated that different treatments had minimal impact on the contents of tea polyphenols and caffeine, with comparable levels among the three. However, in terms of amino acid content, yellow tea treated with pure chemical fertilizer (T0) showed significantly higher levels than those under organic substitution treatments (T1 and T2) (Figure 3A). This aligns with previous research: chemical fertilizers can more effectively increase amino acid content, while organic fertilizers have relatively lower nitrogen utilization efficiency [42,43], resulting in less amino acid accumulation in T1 and T2 compared to T0. As the main component of tea polyphenols, catechins are closely related to the astringency and mellowness of tea infusion. In this study, the total catechin content under T1 treatment was significantly lower than that under T0 and T2. The content of the esterified catechin and EGCG equally showed the lowest levels in T1 compared to T0 and T2, indicating that rapeseed cake fertilizer application attenuated the bitter and astringent taste of yellow tea while enhancing its mellow and refreshing flavor (Figure 3B). This finding aligns with the highest sensory evaluation score for this treatment in the taste assessment (Figure 2B). Theanine is the key contributor to the umami taste of tea. Compared with T0, organic substitution treatments (T1, T2) significantly reduced both theanine content and total umami amino acids, while the levels of sweet umami amino acids, including Asp, Glu, Thr, Ala, Pro, and Met, generally decreased (Figure 3C). Although organic treatment increased sweet amino acids such as Gly and Gln, the total content of umami-sweet amino acids still decreased significantly, which may be one of the important reasons for the overall reduction in amino acid content. On the other hand, the organic substitution treatment also significantly reduced the content of bitter amino acids such as Arg, Val, Ile, Leu, and Lys in yellow tea (Figure 3D), indicating that while reducing umami, it also alleviated bitterness, contributing to a mellow and harmonious overall taste of the tea infusion. A similar finding has been reported in a related research [39]. In summary, although organic substitution fertilization may decrease the content of some umami and sweet amino acids, it significantly reduces bitter amino acids, thereby making the tea infusion more mellow and refreshing in flavor. This result aligns with previous studies.

### 4.3. Consistency Between Sensory Evaluation and the Underlying Key Aroma Compounds

This study, combined with sensory evaluation results (Figure 2), identified differences in aroma scores among various yellow tea treatments: T0 scored 87.5 (slightly tender aroma), T1 scored 86 (relatively high aroma), and T2 scored 89 (distinct tender aroma). Analysis of key aroma compounds using multivariate statistical methods (OPLS-DA, rOAV, and ACI) revealed that the tea’s aromatic characteristics in each treatment were predominantly influenced by the composition and relative contribution of its volatile components. In T0, the primary aroma components were linalool and geraniol, with a combined aroma contribution index (ACI) value of 50.16%, imparting floral and refreshing notes to the tea sample. Previous studies have also indicated that linalool and geraniol were the primary aromatic alcohols in yellow teas, such as Junshan Yinzhen and Huoshan Huangya, and exhibit notable floral characteristics, which is consistent with the findings of this study [44]. However, it also contained a relatively high proportion of δ-cadinene (ACI value 47.90%), which exhibits a sweet, mature aroma [45], partially masks the freshness of the floral notes [46], resulting in an overall aroma that was not particularly outstanding in terms of freshness. The dominant aromatic component of T1 is 1-octanol (ACI value of 56.15%), whose grassy and waxy notes [47] suppress the floral expression of linalool and geraniol (combined ACI of 30.27%). Coupled with the significant contribution of δ-cadinene (ACI value of 12.77%), the overall aroma lacks a bright, refreshing quality, resulting in a slightly lower score than T0. The aromatic profile of T2 tea is primarily determined by linalool (ACI value of 53.5%) and benzeneacetaldehyde (ACI value of 46.5%). Both compounds exhibit floral characteristics, with benzeneacetaldehyde enhancing and accentuating linalool’s floral traits [48]. This combination gives T2 a fresher and more pronounced floral style, earning it higher sensory scores than both T0 and T1. The results above suggest that the overall aroma experience of yellow tea is primarily determined by the composition of key aromatic compounds and their relative contributions. In this study, the rapeseed cake substitution treatment (T1) did not demonstrate superior aroma quality compared to the pure chemical fertilizer control (T0). This finding is inconsistent with some previous research conclusions, which may relate to the processing suitability of the tea variety [49,50]. Previous studies have predominantly focused on green tea systems, whereas different tea types exhibit distinct transformation pathways of aromatic substances during processing and varying flavor standards in final products [51]. This may lead to similar agronomic practices producing different quality effects across various tea categories. Additionally, the T2 treatment (quantum organic fertilizer substitution) significantly outperformed T0 in aroma sensory evaluation, indicating that this fertilization method enhances the aromatic potential of fresh tea leaves, thereby making them more suitable for the development of floral and refreshing PYHT tea products. Future research could further explore the compatibility between fertilizer types and processing techniques, aiming to offer a theoretical basis for targeted high-quality yellow tea production.

### 4.4. Limitations

While this study enhances understanding of the effects of organic substitution treatments on tea sensory quality and quality-related compounds in PYHT teas, several limitations should be acknowledged. A previous study has revealed that organic fertilizer can improve aroma indices, particularly geraniol and linalool [20]. However, our study did not align with this finding (Table 1). The reason for this may be attributed to the different soil properties, which are directly associated with the nutrient cycling for tea plants, thus affecting the metabolism of aroma compounds. One more reason for the inconsistent results could be partially due to the short-term duration of our experiment, which may cause the insignificant effect and shift in the secondary metabolism of tea plants. This limitation underscores the need for long-term observation and larger sample sizes, including soil property variables, in future studies to improve statistical power and robustness. Additionally, although the current surplus in tea production has somewhat diminished the focus on the yield-enhancing effects of fertilizers, tea yield remains a crucial indicator for evaluating fertilizer efficacy and nutrient efficiency, providing valuable insights into the performance of different fertilization treatments. This also highlights opportunities for further refinement in sampling methods and nutrient analysis in subsequent experiments.

## 5. Conclusions

This study demonstrated that varied fertilization treatments significantly affected the aroma quality of PYHT tea, whereas no significant differences were observed in appearance, taste, liquor color, infused tea, or total score. Compared with chemical fertilization, substituting organic fertilizer significantly reduced amino acid content, primarily due to decreases in theanine, arginine, and valine. In contrast, tea polyphenols and caffeine showed no significant differences among treatments. GC-MS combined with multivariate analysis identified five key volatile compounds—octan-1-ol, linalool, geraniol, benzeneacetaldehyde, and δ-cadinene—that differentiated the aroma profiles of the tea samples. The specific abundance patterns of these compounds may contribute to the superior, fresher, and more pronounced floral character of T2 tea, compared with the slightly less fresh floral notes of T0 tea and the stuffy floral expression of T1 tea. In conclusion, these findings offer practical guidelines for fertilizer application in PYHT tea production, which may improve both aroma quality and overall market value. However, while our findings suggest that organic fertilizer amendments, particularly newly applied organic fertilizer, can positively influence specific tea aroma compounds, the mechanisms linking soil properties and nutrient cycling to tea quality remain indirect. These relationships require long-term validation and further investigation. Future research should also prioritize analyzing changes in soil properties and yield effects under different fertilization regimes, as well as conducting multi-year repeated observations, as these represent the primary limitations of the current study.

## Figures and Tables

**Table 1:** rOAVs (above 1) and VIP values (above 1) of differential aroma components among treatments.

NO.	Compounds	OTs ^a^	Concentration (μg/L)			rOAV			ACI		
			T0	T1	T2	T0	T1	T2	T0	T1	T2
1	Octan-1-ol	22.00	nd	182.79 ± 0.88	nd	nd	8.31 ± 0.04	nd	0	56.15%	0
2	Linalool	6.00	16.81 ± 2.26 a	16.92 ± 0.08 a	10.08 ± 0.49 b	2.80 ± 0.38 a	2.82 ± 0.01 a	1.68 ± 0.08 b	22.62%	19.05%	53.50%
3	Geraniol	6.60	22.54 ± 1.45 a	11.05 ± 0.18 b	nd	3.41 ± 0.22 a	1.66 ± 0.03 b	nd	27.54%	11.22%	0
4	Benzeneacetaldehyde	6.30	1.53 ± 0.06 b	0.78 ± 0.00 c	9.17 ± 0.47 a	0.24 ± 0.01 b	0.12 ± 0.00 c	1.46 ± 0.07 a	1.94%	0.81%	46.50%
5	δ-cadinene	1.5	8.89 ± 0.36 a	2.84 ± 0.01 b	nd	5.93 ± 0.24 a	1.89 ± 0.01 b	nd	47.90%	12.77%	0

Note: ^a^ Odor thresholds (OTs) in water and ACIs were calculated by the ratio of the rOAV of volatile compounds to the rOAV of all key volatile components. All values are presented as mean ± SD, and different lowercase letters (a, b, and c) indicate significant differences at *p* < 0.05. ‘nd’ means no detection.

## Data Availability

The original contributions presented in this study are included in the article/Supplementary Materials. Further inquiries can be directed to the corresponding authors.

## Supplementary Materials

The following supporting information can be downloaded at: https://www.mdpi.com/article/10.3390/foods15101655/s1, Table S1: Relative contents of volatile components with VIP value above one.
