# Supplementary material for: Aroma Rather than Taste Quality Exerted a More Pronounced Response to Organic Fertilizer Substitution in a Tea Garden: A Case Study on the Yellow Tea Quality
Source: Foods. 2026 May 9;15(10):1655. doi: 10.3390/foods15101655 (PMC13206336; doi:10.3390/foods15101655)
Supplement: Supplementary file 1 [file foods-15-01655-s001.zip › foods-4223648-supplementary.pdf]

Table S1 Relative contents of volatile components with VIP value above one (μg/g)

CAS number	VIP value	Compound	T0-1	T0-2	T0-3	T1-1	T1-2	T1-3	T2-1	T2-2	T2-3
150-86-7	8.21046	Phytol	363.31	335.02	349.16	47.10	46.65	46.89	10.50	9.55	10.18
111-87-5	7.65512	Octan-1-ol	0.00	0.00	0.00	183.67	181.91	182.79	0.00	0.00	0.00
1191-41-9	5.27359	ethyl (9Z,12Z,15Z)-octadeca-9,12,15-trienoate	154.72	142.67	137.87	31.37	31.07	30.72	0.00	0.00	0.00
100-52-7	2.79168	Benzaldehyde	0.00	0.00	0.00	0.00	0.00	0.00	27.88	25.33	26.61
544-35-4	2.65517	ethyl (9Z,12Z)-octadeca-9,12-dienoate	38.14	35.17	36.65	7.27	7.20	7.24	0.00	0.00	0.00
628-97-7	2.57263	Ethyl hexadecanoate	38.23	35.26	31.30	9.62	9.53	9.58	0.01	0.01	0.01
22258	2.30905	2-Phenylethanol	0.00	0.00	0.00	0.00	0.00	0.00	19.07	17.33	18.20
78-70-6	2.22189	Linalool	18.77	17.31	14.34	17.00	16.84	16.92	10.50	9.55	10.18
106-24-1	2.10926	Geraniol	24.11	22.23	21.27	11.11	11.00	10.76	0.00	0.00	0.00
500-66-3	1.86456	5-pentylbenzene-1,3-diol	0.00	0.00	0.00	10.90	10.79	10.84	0.00	0.00	0.00
73209-42-4	1.61815	(1R,4S)-1,6-dimethyl-4-propan-2-yl-1,2,3,4-tetrahydronaphthalene	0.00	0.00	0.00	0.00	0.00	0.00	9.37	8.51	8.94
122-78-1	1.56028	Benzeneacetaldehyde	1.59	1.46	1.53	0.79	0.78	0.78	9.71	8.83	8.99
38230-60-3	1.44827	(1S,4S,6S,7S,10S)-4,10-dimethyl-7-propan-2-yltricyclo[4.4.0.01,5]dec-8-en-4-ol	11.33	10.45	10.89	2.15	2.13	2.01	0.00	0.00	0.00
119-36-8	1.44749	Methyl salicylate	0.00	0.00	0.00	3.16	3.13	3.14	9.95	9.04	9.50
19912-62-0	1.41835	(1S,4S,4aR,8aS)-1,6-dimethyl-4-propan-2-yl-3,4,4a,7,8,8a-hexahydro-2H-naphthalen-1-ol	11.81	10.89	9.12	2.62	2.60	2.61	0.00	0.00	0.00
39028-58-5	1.3197	(3R,6S)-6-ethenyl-2,2,6-trimethyloxan-3-ol	13.28	12.24	10.32	7.01	6.94	6.74	3.03	2.75	2.89
483-76-1	1.29741	δ-cadinene	9.25	8.53	8.89	2.86	2.83	2.84	0.00	0.00	0.00
96-76-4	1.26307	2,4-ditert-butylphenol	0.00	0.00	0.00	5.00	4.95	4.98	0.00	0.00	0.00
112-39-0	1.23906	methyl hexadecanoate	8.09	7.46	7.77	3.82	3.78	3.80	0.00	0.00	0.00
502-69-2	1.20159	6,10,14-trimethylpentadecan-2-one	7.80	7.19	8.18	3.52	3.48	3.50	0.24	0.21	0.23

73365-77-2	1.14473	(1S,4S,4aS,8aR)-4,7-dimethyl-1-propan-2-yl-2,3,4,5,6,8a-hexahydro-1H-naphthalen-4a-ol	8.11	7.48	8.24	1.72	1.70	1.71	1.82	1.65	1.74
72636-53-4	1.12117	2,5-dibutylfuran	6.20	5.71	5.96	0.00	0.00	0.00	0.00	0.00	0.00
88-58-4	1.09338	2,5-ditert-butylbenzene-1,4-diol	5.89	5.43	5.66	0.00	0.00	0.00	0.00	0.00	0.00
301-00-8	1.07148	methyl (9Z,12Z,15Z)-octadeca-9,12,15-trienoate	6.14	5.66	6.39	1.57	1.56	1.57	0.00	0.00	0.00
31501-11-8	1.05674	[(Z)-hex-3-enyl] hexanoate	2.60	2.40	2.16	4.36	4.32	4.27	7.99	7.26	7.62
79-77-6	1.02707	(E)-4-(2,6,6-trimethylcyclohexen-1-yl)but-3-en-2-one	8.50	7.84	8.17	5.12	5.07	5.09	8.89	8.08	8.48
